# Biologically Inspired Catheter for Endovascular Sensing and Navigation

**DOI:** 10.1038/s41598-020-62360-w

**Published:** 2020-03-27

**Authors:** Erin E. Sutton, Bernhard Fuerst, Reza Ghotbi, Noah J. Cowan, Nassir Navab

**Affiliations:** 1 Department of Mechanical Engineering, Johns Hopkins University, Baltimore, MD United States; 2 Department of Computer Science, Johns Hopkins University, Baltimore, MD United States; 3 Department of Vascular Surgery, HELIOS Klinikum München West, Munich, Germany; 4 Department of Computer Science, Technische Universität München, Munich, Germany

**Keywords:** Interventional cardiology, Medical imaging, Biomedical engineering

## Abstract

Minimally invasive treatment of vascular disease demands dynamic navigation through complex blood vessel pathways and accurate placement of an interventional device, which has resulted in increased reliance on fluoroscopic guidance and commensurate radiation exposure to the patient and staff. Here we introduce a guidance system inspired by electric fish that incorporates measurements from a newly designed electrogenic sensory catheter with preoperative imaging to provide continuous feedback to guide vascular procedures without additional contrast injection, radiation, image registration, or external tracking. Electrodes near the catheter tip simultaneously create a weak electric field and measure the impedance, which changes with the internal geometry of the vessel as the catheter advances through the vasculature. The impedance time series is then mapped to a preoperative vessel model to determine the relative position of the catheter within the vessel tree. We present navigation in a synthetic vessel tree based on our mapping technique. Experiments in a porcine model demonstrated the sensor’s ability to detect cross-sectional area variation *in vivo*. These initial results demonstrate the capability and potential of this novel bioimpedance-based navigation technology as a non-fluoroscopic technique to augment existing imaging methods.

## Introduction

## Clinical Motivation

The number of vascular procedures performed under fluoroscopic guidance is increasing as minimally invasive techniques are developed for the diagnosis and treatment of a widening array of vascular diseases^1,2^. With the reliance on fluoroscopic imaging for device guidance and navigation comes increased radiation dose to the patient^3^ and interventional staff^4–6^. Furthermore, these complicated endovascular procedures require dynamic navigation and accurate positioning of interventional devices relative to the vascular tree, which can be extremely challenging with only two-dimensional fluoroscopic images (see Supplementary Material for example case). As a result, multiple contrast agent injections, image acquisitions, and catheter exchanges are often necessary.

There are existing techniques to reduce radiation use during endovascular procedures. Systems such as CARTO 3 (Biosense Webster, Diamond Bar, CA) and Rhythmia (Boston Scientific, Marlborough, MA) employ external electromagnetic or external electric sources on the outside of the body to localize catheters in the cardiac chamber. These technologies have revolutionized electroanatomic mapping of the heart and reduced radiation exposure during cardiac electrophysiology procedures^7–10^ but are currently unsuitable for catheter navigation through the vessel tree. In fact, the catheters are usually navigated from the femoral artery using fluoroscopic guidance, because the sensing volume created by the external sources is limited. The accuracy of the position estimate is greatly diminished in the presence of patient movement, vessel deformation, and unstable heartbeats^11,12^. Additionally, these techniques require specialized large-diameter electrophysiology catheters, are incompatible with many intravascular devices (e.g. stents), and necessitate significant changes to the surgical workflow. The medical imaging community, including our group, has focused on registration of pre-interventional images to live 2D interventional images to track catheters and guidewires^13–17^. Pre-interventional imaging such as computed tomography angiography (CTA) and magnetic resonance imaging are commonly used for diagnosis of vascular disease such as aneurysms or blockages, and registration techniques attempt to leverage the rich 3D model to enhance the 2D interventional image. However, these techniques exhibit substantial error caused by unavoidable vessel deformation as devices are inserted and manipulated^16,18^. Furthermore, these image processing solutions still rely on fluoroscopic image acquisition.

**Figure 1:**
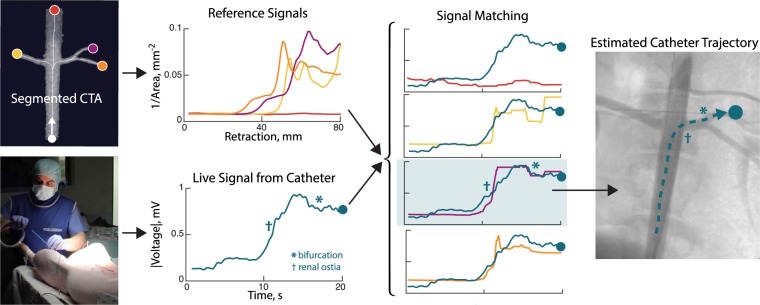
Schematic of Bioelectric Navigation. The current state-of-the-art for endovascular navigation requires multiple x-ray images and contrast injections to generate a view of the catheter inside the blood vessels, as shown in the right image. In our method, a bioelectric catheter detects anatomical landmarks like ostia and bifurcations. Software registers the live bioelectric measurements from the catheter to simulated signals from a pre-interventional image to determine the catheter’s position in the vascular tree. This technique could augment fluoroscopic guidance, providing three-dimensional information, the correspondence of the impedance variation with the pre-interventional image, without additional imaging or contrast.

To navigate to an area of interest, the interventionalist primarily uses contrast-enhanced fluoroscopic images to estimate the current position of the catheter. However, the exact 3D position and orientation of the catheter is not necessary for its navigation, as long as the interventionalist knows the catheter’s current branch and the path to the area of interest. Therefore, we propose a system in which a measure of the catheter’s immediate surroundings is used to identify what branch contains the catheter and how far the catheter has traveled into the branch (Fig. 1). In principle, a sensor directly on the catheter tip could be used to detect features of the local vessel geometry without the need for image registration or external sensors. Optical coherence tomography (OCT) uses one such sensor-equipped catheter. Originally developed to image transparent tissues in the eye^19^, it has since been applied to the *in situ* classification of intravascular plaque^20^. However, OCT uses a fast, automated pullback to image the cross section of very short vessel segments and requires flushing of the vessel with saline, rendering it unfeasible for navigation^21^. Another commercially available sensor technology is intravascular ultrasound (IVUS), which has been used alone^22^ and with OCT^23^ to image plaque and verify stent apposition. The registration of IVUS images with angiography has been attempted, but the most salient structure visible in an IVUS image is plaque, which is not generally visible in CTA, so the local measurements could not be accurately mapped back to the pre-interventional 3D model^24^.

## Bioinspiration

Here we introduce a technique inspired by electric fish that incorporates measurements from a newly designed electrogenic sensory catheter with preoperative imaging to provide continuous feedback during vascular procedures without additional ionizing radiation, contrast injection, image registration, or external tracking. For inspiration, we looked to the weakly electric fish^25^. This fish has an electric organ that discharges a signal, generating a weak electric field around its body. The electrical current flowing through the epidermis of the fish allows the measurement of the distribution of amplitude and phase across the body surface, which is referred to as the “electric image”^26^. If an object is within the range of propagation of the signal, it influences the field and the object is detectable via change in the electric image, depending on the electrical properties of the object^25^. Studies have shown that weakly electric fishes not only consider the intensities of the responses but also the modulation on the surface, the normalized modulation and the slope-to-amplitude ratio to detect the distance^27,28^, material, size^29^ and electrical properties^30^ of surrounding objects individually. The fish even modulate the electric organ discharge to improve object detection and image quality^31^.

## Impedance measurement

Like the fish, our sensorized catheter measures the changes to its self-generated electric field to sense its local surroundings. Our first prototype includes ring electrodes distributed near the catheter tip. The electrodes simultaneously create a weak electric field and measure the impedance. The impedance of blood is much lower than that of vessel walls and surrounding tissue^32^, so the catheter detects local vessel geometry (bifurcations, stenoses, aneurysms) from measured impedance. A simplified model of the impedance in a cylindrical vessel is:

1 $${Z}_{T}={\left(\frac{A\sigma }{L}+{G}_{p}\right)}^{-1}$$

The blood conductivity, *σ*, is assumed constant throughout the duration of a procedure, *A* is the cross sectional area of the vessel, and *L* is the distance between the electrodes. Critically, the parallel conductance through surrounding tissue, *G*_*p*_, is proportional to cross-sectional area, *A*^33^. This relationship implies that the impedance varies inversely with vessel cross-sectional area. Physics-based numerical simulations with relevant vessel and electrode geometries confirmed the predictions of this simplified model (see Results). Our central hypothesis is that as the electrodes passes a bifurcation, restriction, aneurysm, or other local change in the vessel cross-sectional area, the catheter will detect a significant disturbance to the electric field. The bioelectric signal represents a distinctive profile of the path taken through the vessel tree.

The navigation system we developed relies on a custom electronic system for signal generation, recording, and conditioning. Vascular impedance measurement has been proposed for blood constituent monitoring^34^, lumen sizing^35,36^, aortic segmental volume measurement^37^, and lesion classification^38,39^. Like IVUS and OCT, these methods rely on fluoroscopic imaging for catheter guidance. The current work addresses a critical barrier to using bioimpedance as a non-fluoroscopic alternative for catheter guidance in the vessel tree: matching the measured impedance signal to the device’s location in vascular tree.

## Modeled and Empirical Signal Matching

Our electrogenic catheter measures the dynamic bioelectric signals as it moves through the vessel tree. By identifying the path corresponding to the real-time signal from the catheter, our software informs the interventionalist of the branch most likely containing the catheter and the catheter’s position relative to relevant anatomical landmarks in the vascular tree. In this fashion, our system bridges the gap between catheter-based sensing and catheter navigation. The local voltage measurement from the catheter is compared to predicted “reference” measurements derived from a pre-interventional anatomical model (e.g. obtained from a segmented CTA as in our work). Thus, our system is designed to identify the global position of the catheter relative to the vessel tree.

A bioimpedance signal obtained from the catheter as it is advanced through the vessel tree is a scaled and time-warped version of the vessel cross-sectional area along the catheter path. The alignment of measured bioimpedance from the catheter with the pre-interventional model of the vessel tree is the foundation of our technique. As a proof of concept, our experiments use open-ended dynamic time warping (OE-DTW)^40^ to perform this alignment. OE-DTW was chosen because it can be adapted to provide feedback to the interventionalist during a procedure, and it includes a similarity measure that enables the determination of the most likely position of the catheter with respect to the vessel model. Additionally, OE-DTW enables the alignment of incomplete test time series with complete references. Ideally, the incomplete voltage series during a procedure is incrementally compared to each of the complete references from the model to obtain constant feedback about the predicted location of the catheter. In our implementation, cross-sectional area along each path forms the reference dataset, and the experimentally measured voltage magnitude during catheter navigation is the test time series.

## Results

### Computational validation of impedance model

**Figure 2:**
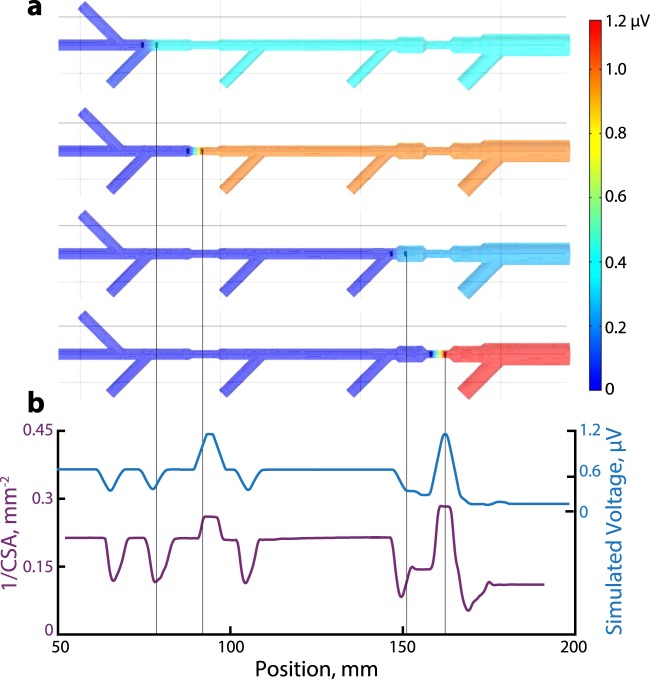
(**a**) The reference measurements are simulated in a synthetic vessel phantom from imported CAD geometry. The electrodes (black) at four different positions are rendered within the vessel geometry. The colors indicate the electric field relative to the left-most electrode. The voltage decreases at a bifurcation and increases at a stenosis. (**b**) The simulated voltage magnitude (blue) and the inverse of the cross-sectional area (purple) from the segmented CBCT agree.

A complete bioimpedance model requires solution of the 3D Poisson equation, assuming known permittivities of blood and tissue. Given a relatively simple geometry, one can employ finite element analysis to numerically solve for the electric potential distribution. For our first feasibility experiments, we designed an eight-path vessel phantom with two stenoses and one aneurysm. We imported the 3D CAD model into Comsol Multiphysics (COMSOL, Inc., Stockholm, Sweden) and simulated the signal as a two-electrode catheter passed through the six primary branches (Fig. 2a). The simulation yielded six distinct models, one for each path. The simulated voltage at the emitting electrode was inversely proportional to the cross-sectional area extracted from the cone-beam CT (CBCT) (Fig. 2b). We conclude that cross-sectional area is adequate for localization with two ring electrodes on the catheter tip, the minimum required for bioelectric sensing and navigation. The preliminary prototype used in the following experiments had one emitting electrode and one sensing electrode.

The simulation enabled the modeling of difficult, but realistic, scenarios such as the influence of contact with a vessel wall or the presence of stents. We require that the catheter be able to detect a branch significantly smaller than then primary vessel where the branch occurs, and this simulation framework provides the means to modify the acquisition strategy to achieve this goal. In particular, the simulation framework we developed enables the efficient evaluation of changes to the localization system (e.g. signal modulations, electrode configurations, catheter diameters). With this system, we determined that a 6F (2 mm diameter) catheter could detect ostia of approximately 2.5 mm diameter from a 10 mm main vessel. The 6F prototype was chosen for convenience, and we have begun to miniaturize the sensor with a custom guidewire for smaller vessels.

### Synthetic vessel tree navigation

**Figure 3:**
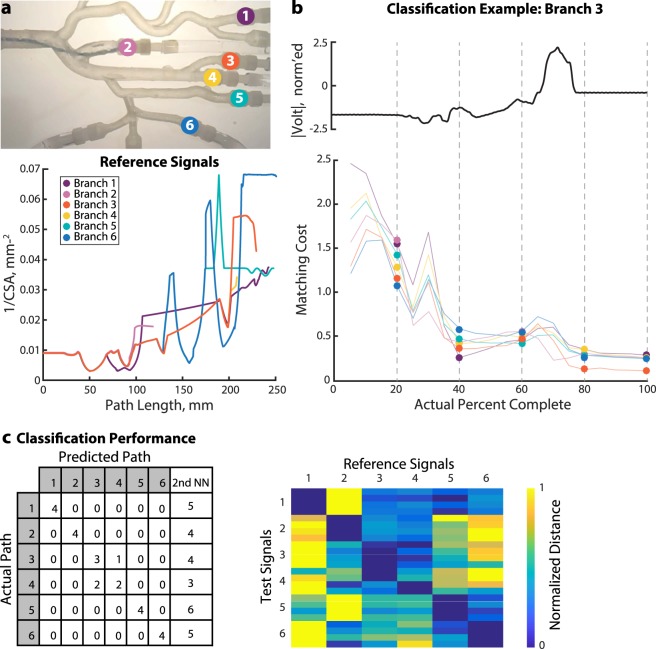
Navigation in Anatomical Phantom. (**a**) Phantom with six paths marked. The catheter was manually advanced from the trunk on the left side of the photograph into the branches on the right. The catheter is in Branch 2 in this image. Below, the reference signals for this experiment were the inverses of the cross-sectional areas extracted from the CAD geometry. Each trough represents a bifurcation or widening, and each peak indicates a stenosis or narrowing. (**b**) In this example, the catheter traveled through Branch 3. As it progressed, the matching algorithm computed a matching cost between the incomplete test signal and all possible lengths of each reference signal. The branch with the lowest cost was selected as the most likely location of the catheter. (**c**) The matching algorithm correctly classified the test path for 21/24 trials. Test paths 3 and 4 were misclassified. On the right, normalized distance measure *D*_*ij*_ is given for each of the 24 trials. Blue indicates a low distance, corresponding to a low matching cost and high similarity between the test and reference signals. Yellow indicates a larger distance between the test and reference.

To validate the relationship between impedance and cross sectional area in a more complex vascular geometry and implement our navigation algorithm, we have designed and 3D printed an acrylic phantom of major branches of the femoral and left coronary arteries. Prior to navigation, we compute the cross-sectional area along the centerlines of the six paths of the phantom reachable with our electrophysiology catheter (see Methods). These unique cross-sectional area profiles represent the possible paths of the phantom and serve as the dictionary of reference signals for signal matching (Fig. 3a).

We connected the catheter to a constant current source and a low-power bioelectric signal acquisition system. As the catheter was manually advanced through each of the six branches of the phantom, the system recorded a unique voltage magnitude signal for each branch. Post hoc analysis of a simultaneously recorded video confirmed the catheter’s trajectory during each trial. An example test signal and associated matching is shown in Fig. 3b.

To the quantify the similarity between a given test signal and all reference signals, we used the OE-DTW distance measure, *D*_*ij*_, between the test signal *i* and reference *j*. *D*_*ij*_ approximates the insertion-length-normalized sum of absolute errors (see^40^ for details). For visualization purposes (Fig. 3c) we normalized this distance measure for all test signals for a given reference, i.e.

$${\bar{D}}_{ij}=\frac{{D}_{ij}-{{\rm{\min }}}_{j}{D}_{ij}}{{{\rm{\max }}}_{j}{D}_{ij}}$$

For complete test signals, the algorithm correctly classified the test path in 21/24 trials (Fig. 3c). When it failed, the second-best match was consistently the correct path; the correct classification was always in the algorithm’s top two choices for best match. This ambiguity arose in these specific cases because two paths with substantial overlap are not well distinguished. For instance, only the final 5% differ between Path 3 and Path 4, so in practice the catheter would need to advance further to acquire a sufficiently discriminating BSN signal. As an example, consider Paths 5 and 6. Immediately following the fourth bifurcation, the reference signals for Path 5 and 6 are nearly identical (with only a slight diameter difference). The ambiguity is resolved once the catheter encounters either the stenosis on Path 5 or the fifth bifurcation on Path 6.

### *In vivo* renal artery detection

The heterogeneity of whole blood, blood flow, and electrical noise associated with the operating room may complicate bioimpedence-based sensing in clinically realistic settings. The objective of our initial *in vivo* study was to test whether the catheter tracking system was capable of detecting vascular branches, specifically the renal arteries.

Bioelectric recordings were performed in a single live domestic swine (*sus scrofa domestica*). The left renal artery was accessed from the right femoral artery and a sheath was placed at the access point. Under fluoroscopic guidance, the surgeon navigated the catheter to the infrarenal abdominal aorta. An angiographic roadmap of the area of interest was obtained. The bioelectric sensing system acquired signals that were synchronised with fluoroscopic images as the surgeon advanced the catheter into the suprarenal aorta, left main renal artery, and left superior as well as inferior segmental renal artery.

**Figure 4:**
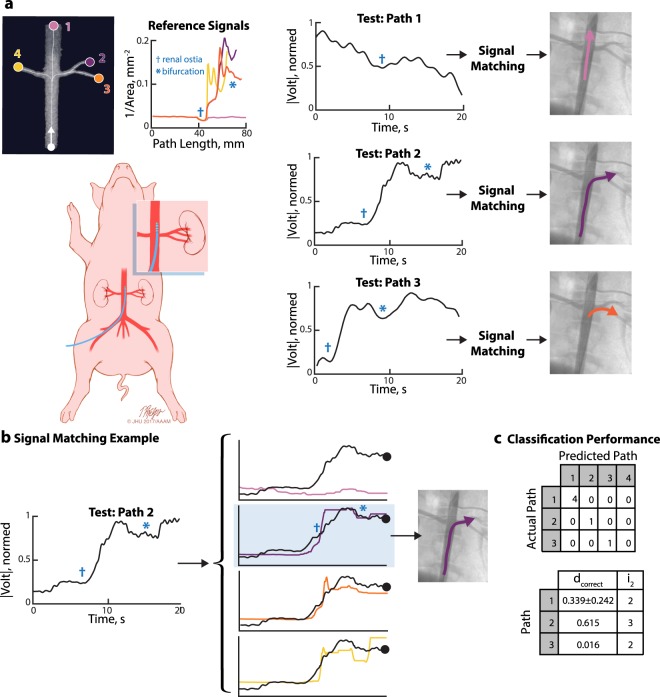
*In vivo* renal artery navigation. (**a**) Before the surgical procedure, the segmented CTA was processed to extract the reference signals (inverse cross-sectional area). The catheter was not advanced down the right renal artery (yellow, Path 4), but it was included as a reference signal to test if the system could distinguish between the left and right renal arteries. Permission is granted for one time use in the publication in all languages and all media. The figure is not to be separated from the article and may not be altered. The figure is not covered by the CC BY license. Illustration: Tim Phelps ©2019 JHU AAM, Department of Art as Applied to Medicine, The Johns Hopkins University School of Medicine. (**b**) OE-DTW takes in a test signal and set of reference signals. The test signal (from Path 2 here) is matched against warped versions of the reference signals, and the warping with the lowest cost is selected as the most likely classification. (**c**) All trials were correctly classified.

The BSN system behaved as expected and successfully detected the renal artery ostia and several branches of the left renal artery (Fig. 4). While there was increased high frequency noise compared to previous experiments, it did not substantially affect the detection of branches. In fact, the matching algorithm successfully classified all trials in this experiment. Even this prototype system discriminated between the left renal and right renal arteries, which suggests that the slight narrowing at the opening of the left renal artery was sufficient for our system to distinguish the two paths.

## Discussion

A system that measures geometry changes from inside a vessel in real-time could improve patient and physician safety during common procedures. The system proposed in this paper relies on pre-interventional images to estimate the cross sectional area of vessels en route to a surgical site, and then matches a sequence of interventional bioelectric signals to the pre-interventional model based on the inverse relationship between impedance and cross sectional area. Non-fluoroscopic navigation with BSN was demonstrated in simulation and in an anatomical phantom. Bifurcation detection and artery discrimination with BSN were validated in a live animal test.

In the phantom study, similar trials were misclassified (Paths 3 and 4). Clinically, this result indicates that it is difficult to distinguish between two branches of a vessel unless the catheter travels a considerable distance down either branch. However, this is not expected to negatively affect the clinical workflow. In the clinical state-of-the-art, catheter tracking with fluoroscopy, clinicians routinely employ a “guess and check” method of advancing the catheter, injecting contrast, checking its position on a fluoroscopic image, and re-positioning the catheter. Our algorithm requires the same movement of the catheter, but without relying on x-ray acquisition for feedback.

The initial, narrowly focused *in vivo* experiment was instrumental in showing that the system detects branches in a clinically relevant setting. In particular, strong correspondence between the interventional voltage and the pre-interventional model geometry suggests that the interventional signal from the catheter is suitable for navigation *in vivo*. Even the simple preliminary prototype could be employed as an augmentation to fluoroscopic catheter navigation by detecting bifurcations in real time without contrast injection or additional imaging. These results are promising, and a more comprehensive *in vivo* study of bioelectric signals and their mapping to a pre-interventional model for a larger region of interest will pave the way for preclinical implementation.

## Outlook

The next challenge we will address is the real time implementation of a matching algorithm and its validation through systematic endovascular navigation in a live animal. In our prototype system, matching is uncertain when the distance between a test and two candidate references is small. In such cases, we envision our real time algorithm to prompt the clinician who can decide whether to continue insertion until unambiguous impedance landmarks are acquired by the BSN sensor or take a fluoroscopic image to confirm the position of the catheter. The interventionalist can verify the position of the catheter, and our algorithm will use the position measurement to inform subsequent position estimates.

The generation and measurement of bioelectrical signals within vessels and their mapping to a patient-specific vessel model has never been proposed for endovascular navigation, and it could have an significant near-term clinical impact. For instance, a simple two-electrode catheter like the one used in our experiments could provide feedback during central venous catheter or umbilical catheter placement, thereby improving safety and accuracy in interventions usually performed without guidance. In the long term, bioimpedance-based navigation has the potential to reduce the dependence on fluoroscopy in many common endovascular procedures and, in turn, reduce radiation exposure to patients and interventionalists.

## Methods

### Navigation in anatomical phantom

#### Signal generation and measurement

A function generator supplied a sinusoidal signal to the current source, and the current source supplied a constant *μ*A-scale signal to the emitting electrode on the catheter. A neighboring electrode was grounded. The signal between the two electrodes was amplified and filtered by a low-power bioelectric signal acquisition system (RHD2000, Intan Technologies, Los Angeles, USA). The Intan software (Intan Interface 1.4.2, Intan Technologies, Los Angeles, USA) logged the AC voltage measurement from the electrodes at 25 kHz and filtered the signals. The signal was converted into the frequency domain by a sliding window discrete Fourier transform, and the magnitude at the input frequency was extracted for each window. In this fashion, the input signal enabled relatively simple signal identification. Although real-time implementation is crucial to navigation, all of these experiments involved only post-hoc analysis.

#### Reference signal acquisition

**Figure 5:**
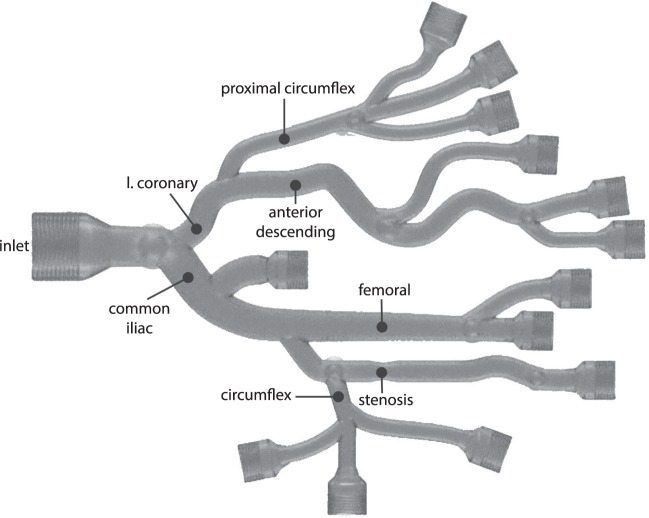
Rendering of 3D-Printed Anatomical Phantom. In this image, the top half of the phantom is modeled after the branches of the left coronary artery. The bottom half mimics the branches of the external iliac artery. The entire phantom measures 22 cm × 16 cm × 4 cm, and the interior diameters are 3–10 mm. For durability, we coated the exterior of the phantom in clear epoxy.

This experiment was conducted in a custom 3D-printed (Multi Jet Fusion process) UV-cured acrylic phantom (Fig. 5). This phantom was designed with anatomically relevant branching, cross-sectional area (3–12 mm), and tortuosity. It has threaded inlets and outlets to enable easy connection to tubing. Furthermore, the phantom is designed to interface with the same components used in the operating room: Luer-lock fittings, guidewires, and introducer sheaths. The phantom is filled with 0.9% saline solution and a catheter is introduced through a sheath.

Before the experiment, we computed the cross-sectional area along the centerlines of six paths of the phantom. These unique cross-sectional area profiles representing the possible paths of the phantom served as the reference signals for the signal matching algorithm (Fig. 3a).

#### Experimental procedure

To navigate through the tortuous vessels, we used a steerable commercially available cardiac electrophysiology catheter (ViaCath 5F, Biotronik, Berlin, Germany). Its ten ring electrodes are 2 mm wide with 5 mm spacing. The input electrode was the second electrode from the distal end, and the next most proximal electrode was grounded. A camera recorded the trajectory of the catheter through the phantom as it was manually advanced through six paths at 1–2 mm/s. Four acquisitions were performed for each path. Four replicates were performed for each path for a total of 24 trials.

### Matching validation *in vivo*

#### Signal generation, measurement, and analysis

We used a 6F cardiac electrophysiology catheter (MultiCath 10J, Biotronik, Berlin, Germany). Its ten ring electrodes were 2 mm wide with 5 mm spacing. Again, the input electrode was the second electrode from the distal end, and the next most proximal electrode was grounded. The input to the current source was  ±5 mV at 730 Hz, and the current source supplied a constant 18 *μ*A to the emitting electrode. A current of this magnitude are well below the acceptable low frequency leakage current, is considered safe, and is not sufficient to stimulate nerves or muscles. A digital oscilloscope (PicoScope2203, Pico Technology, Cambridgeshire, United Kingdom) recorded the voltage from the catheter. Signals were analyzed as in the previous section.

#### Reference signal acquisition

A full body CT with angiography scan of the sedated animal was obtained using a clinical CT machine. The abdominal aorta neighboring the renal arteries was segmented using commercial software (VMTKLab 1.5.4, Orobix, Bergamo, Italy). Segmentation of the vascular tree took approximately one hour. Custom software computed the cross-sectional area along the centerlines of four paths of the segmented model. The set of inverse cross-sectional areas was the reference set for this experiment (Fig. 4a).

#### Surgical procedure

This animal study was conducted at Klinikum Rechts der Isar in Munich, Germany in accordance with relevant guidelines and regulations. The experimental protocols were approved by the Bavarian Government Veterinary Office. Animal care and use was performed by qualified individuals supervised by clinical veterinarians. A single female German country pig (body weight 72 kg) was used for *in vivo* validation of bioelectric sensing.

The animal was sedated with an intramuscular injection of TKX at a dose of 1 cc/50 lb. After sedation, an intravenous catheter was placed in the animal’s ear vein and the animal was intubated to maintain an open airway. General anesthesia was maintained with isoflurane (0.5–5.0%) with oxygen supplementation, and the animal was mechanically ventilated for the duration of the imaging and experiment. The femoral artery was located, prepared, punctured and cannulated. An angiographic roadmap of the area of interest was obtained. The 6F bioelectric catheter was inserted through a sheath, the tip of which was positioned in the infrarenal abdominal aorta under fluoroscopic guidance. Bioelectric signals were collected simultaneously with fluoroscopic images as the catheter was manually advanced through the sheath into the suprarenal aorta, left main superior renal artery, and left left superior as well as inferior segmental renal artery 1–2 mm/s. The ostium of the left renal artery was detected correctly by bioeletric signals. After inserting the bioelectric catheter in the left renal artery, the anatomy including inferior segmental renal artery could be detected using the aqcuired bioelectric signals. The endovascular expert then verified the results based on angiographic imaging. At the end of the experiment, euthanasia of the anesthetized animal was carried out via anesthetic overdose.

## Supplementary information

Supplementary Information.

## Data Availability

The datasets generated and analysed during the current study are available in the Johns Hopkins University Data Archive at 10.7281/T1/4F90KQ.
