# Supplementary figures and images for: Biologically Inspired Catheter for Endovascular Sensing and Navigation
Source: Sci Rep. 2020 Mar 27;10:5643. doi: 10.1038/s41598-020-62360-w (PMC7101317; doi:10.1038/s41598-020-62360-w)

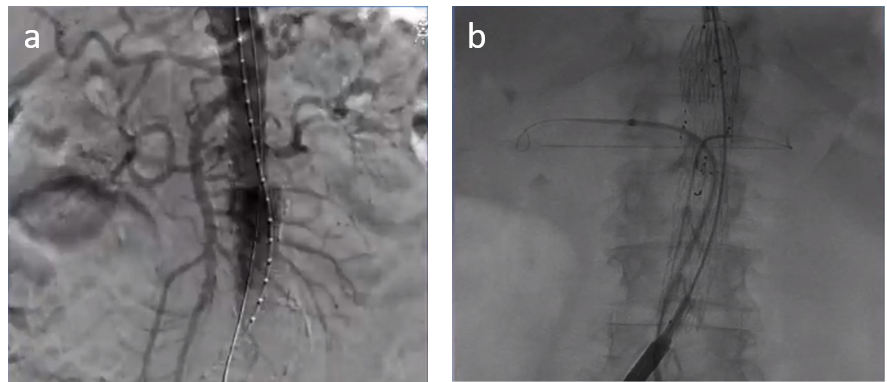

Supplement: Supplementary file 1 — Supplementary Information. [file 41598_2020_62360_MOESM1_ESM.zip › figs/S_renal.png]

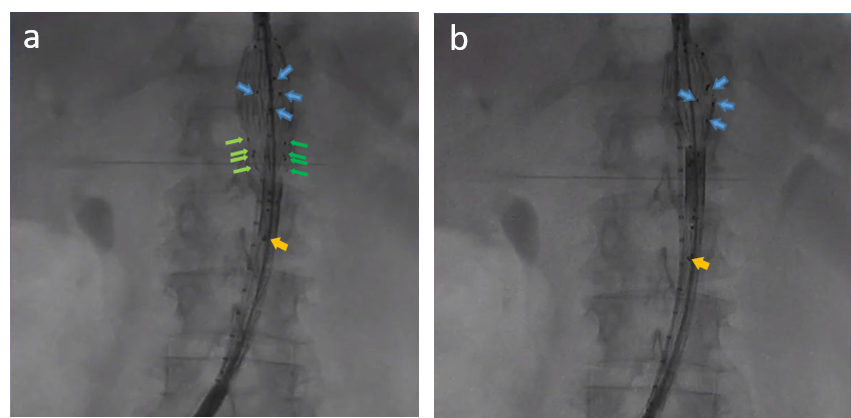

Supplement: Supplementary file 1 — Supplementary Information. [file 41598_2020_62360_MOESM1_ESM.zip › figs/s_repositioning.png]
